# Cardiac Inotropic Effect of Long-Term Administration of Oral Thymoquinone

**DOI:** 10.1155/2019/8575136

**Published:** 2019-06-25

**Authors:** Lubna Ibrahim Al Asoom, Mohammad Taha Al-Hariri

**Affiliations:** Physiology, College of Medicine, Imam Abdulrahman Bin Faisal University, P.O. Box 2114, Dammam 31541, Saudi Arabia

## Abstract

**Back Ground:**

Long-term administration of* Nigella sativa* showed cardiac hypertrophic and positive inotropic effects. Thymoquinone (TQ) is an active ingredient in* Nigella sativa*. Therefore, we aimed to test the cardiac effects of long-term TQ administration.

**Materials and Methods:**

Twenty adult Wistar rats weighing (150-250 g) were divided into two groups: control and TQ. A TQ-olive oil solution was administered orally to the TQ group (dose 10 mg/kg) for two months. An equivalent volume of olive oil was given to the control group. Langendorff isolated hearts were studied. Peak tension, time to peak tension, half relaxation time, and myocardial flow rate were determined. Heart and left ventricle weights and ratios were recorded.

**Results:**

The TQ group exhibited significantly higher peak tension than the control group. There were no significant differences between the two groups in time to peak tension, half relaxation time, and myocardial flow rate. Likewise, there were no signs of cardiac hypertrophy.

**Conclusions:**

Long-term administration of oral TQ induced a positive inotropic effect in the form of an increase in peak tension. TQ administration did not result in cardiac hypertrophy or an increased cardiac metabolic demand at the studied dose. TQ may be a promising inotropic agent.

## 1. Introduction

Heart failure is a major health problem. It is estimated to affect approximately 38 million people worldwide, and this number is expected to increase exponentially due to the current modern life style [1, 2]. In the United States, the prevalence of heart failure was 8.3% in 2015 [3]. In Asia, the prevalence ranges from 1-3% in different countries [4]. Providing proper health care for the management and hospitalization of affected individuals creates an economic burden in many countries [5]. In the US, the medical cost of heart failure is approximately 32.4 billion annually [5].

The current management of heart failure involves reducing signs and symptoms and enhancing cardiac contractility. However, the current available therapeutic strategies fail to prevent the progression of heart failure [6]. Furthermore, the inotropic agents used to augment cardiac contractility, including digoxin, catecholamines, and dopamine, are limited by their undesirable effects [7, 8], such as tachycardia and increased metabolic demand, which could consequently lead to cardiac ischaemia and arrhythmias [9]. Therefore, there is a growing need for new inotropes that are economical and medically safe.

Thymoquinone (TQ) is an active ingredient in* Nigella sativa *Linn, which is traditionally known in Middle Eastern countries as Black seed.* Nigella sativa* (*Ns*) seeds consist of 36-38% fixed oil, protein, alkaloids, and saponin. In addition, 0.4-0.45% of* Ns* seeds is essential oil, which is characterized by its major constituent, TQ [10].

*Ns* and TQ show a wide range of pharmacological and therapeutic actions as reported sporadically in the literature, including anti-inflammatory [11], analgaesic [12], antioxidant [13], hypoglycaemic [14], and lipid-lowering effects [15].

Few favourable cardiovascular therapeutic effects have been reported for* Ns* and TQ. In a double-blinded randomized controlled study,* Ns* showed a blood pressure-lowering effect in both systolic and diastolic blood pressure [16]. Similarly, the administration of isolated TQ elicited a hypotensive effect in L-NAME-treated adult Wistar rats [17]. In addition, both* Ns* and TQ showed evidence of protective effects against ischaemia and reperfusion when administered acutely or chronically to Wistar rats [18, 19]. Furthermore, long-term administration of* Ns* resulted in a significant increase in the expression of antigens and growth factors promoting coronary angiogenesis [20]. The inotropic effect of long-term* Ns* administration was described by el-Bahai et al. in the form of increases in peak tension and the rate of force development in adult rats [21]. The same group also found cardiac hypertrophy after* Ns*-treatment for two months [22]. However, there are no previous reports on the possible inotropic effect of long-term TQ administration. TQ may induce the cardiac effect of* Ns*. Therefore, in the current study, we aim to determine the effects of long-term administration of oral TQ for two months on the hearts of adult Wistar rats and to compare our findings to the effects of long-term* Ns* administration.

## 2. Materials and Methods

### 2.1. Animals

Twenty healthy adult Wistar albino male rats were obtained from the animal house of the Center of Research and Medical Consultation Studies, Imam Abdulrahman Bin Faisal University (formerly known as University of Dammam). Their weights ranged from 150 to 250 g. The animals were housed individually in labelled cages at a controlled temperature of 22°C and a 12-h light/dark cycle, and they had free access to water and laboratory chow. Body weight measurements were performed weekly for each rat to adjust the oral dose of thymoquinone. The study was performed according to the institutional rules of Imam Abdulrahman Bin Faisal University regarding animal experiments. Ethical approval was provided by the ethical committee of the deanship of scientific research in Imam Abdulrahman Bin Faisal University with reference number (IRB-2014-01-165). This committee is a branch of the national committee of Bioethics, Saudi Arabia (http://bioethics.kacst.edu.sa/).

### 2.2. Thymoquinone and Olive Oil

TQ was obtained from SIGMA-ALDRICH, USA (product No. 274666) and had a purity of 98.5%. The TQ-olive oil solution was prepared by dissolving 100 mg of TQ powder in 10 ml of olive oil. Olive oil was purchased from the market (originally a product of Italy and packed for Sasso via Benvenuto Cellini 75, Tavarnelle Val di Pesa, Italy). The solution was mixed vigorously before use and had a concentration of 10 mg/ml TQ.

### 2.3. Study Protocol

#### 2.3.1. The Effect of Long-Term Administration of TQ

The rats were divided randomly into two study groups (A and B), each containing 10 rats. Group A received a daily oral dose of 10 mg/kg TQ for two months. This dose was equivalent to the submaximal dose of* Ns* that produces inotropic and hypotensive effects in rats [21, 23] and similar to the dose used to inhibit isoproterenol-induced myocardial injury [24]. Group B was the control group. The volume of the TQ solution in olive oil needed to supply the required dose of TQ was given to the experimental group daily through orogastric feeding. An equivalent volume of olive oil was administered to the control rats. Treatment was given to all rats in the morning between 8 and 9 am.

### 2.4. Isolated Perfused Heart Preparation

After two months of oral TQ supplementation, each rat was weighed, injected intraperitoneally with 5000 IU of heparin sodium, and anaesthetized with phenobarbital (40 mg/kg body weight). Through a thoracic incision, the heart was excised and mounted via Langendorff preparation (Langendorff apparatus, AD instruments, Australia). Hearts were perfused via Langendorff preparation as described previously [25]. The perfusion fluid was a modified Krebs-Henseleit bicarbonate buffer of pH 7.4 equilibrated with O2:CO2 (95:5) at 37°C and containing the following (in mM): NaCl: 118, KCl: 4.7, CaCl2 2.5, MgSO4 1.2, KH2PO4 1.2, NaHCO3 25, Na2EDTA 0.5, and dextrose 11. Isolated perfused heart preparations were allowed to stabilize for 15 minutes prior to recording the baseline values of myocardial flow rate, peak tension (PT), time to peak tension (TPT), and half relaxation time (HRT). TPT was measured as the time from the onset of a contractile response to the peak of developed tension. HRT was measured as the time required for tension to fall from its peak to 50% of the response.

### 2.5. Heart Weight

Hearts were freed from fat and fibrous tissue and dried with filter paper before determining the weights of the whole heart and the left ventricle. Cardiac indices were calculated as ratios of whole heart weight/body weight (HW/BW), left ventricular weight/body weight (LVW/BW), and left ventricular weight/whole heart weight (LV/HW).

### 2.6. Data Analysis and Statistics

For the statistical evaluation of the results, Statistical Package for Social Sciences (SPSS) version 20 for Windows was used. All data were expressed as the mean ± SD. An unpaired t- test was run to determine differences between the experimental and control groups. A P-value <0.05 was considered statistically significant.

## 3. Results

**Figure 1:**
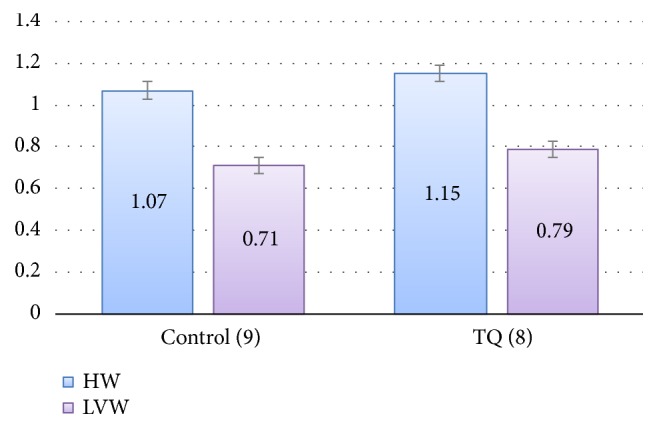
Comparison of heart and left ventricle weights (HW, LVW) (g) of the control and thymoquinone groups. TQ: Thymoquinone, HW: heart weight, LVW: left ventricular weight.

**Figure 2:**
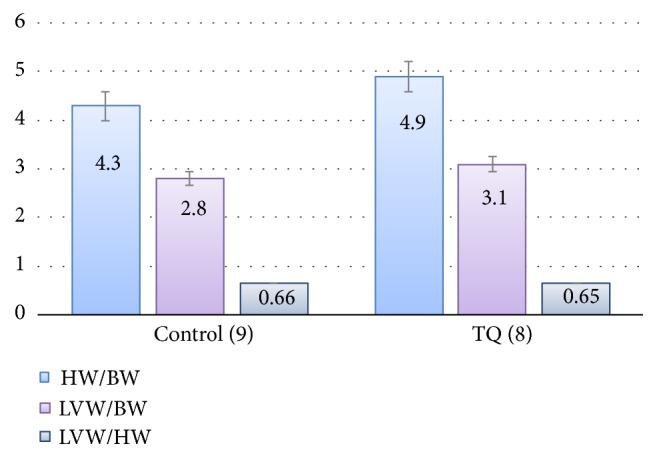
Comparison of HW/BW, LVW/BW, and LVW/HW ratios of the control and thymoquinone groups. TQ: thymoquinone, HW: heart weight, BW: body weight, LVW: left ventricular weight.

The initially recruited rats for the two groups were homogenously matched for body weight. By the end of the study, there were 8 rats in the TQ group and 9 rats in the control group. The other rats died due to trauma caused by the orogastric needle. There was no significant difference between the two groups in final body weight, actual weight gain, or the percentage of weight gain (Table 1). No evidence of cardiac hypertrophy was found in the two groups. Heart weights, left ventricular weights (Figure 1), HW/BW, LVW/BW, and LVW/HW (Figure 2) showed no significant differences between the TQ and control groups using unpaired t-tests.

**Figure 3:**
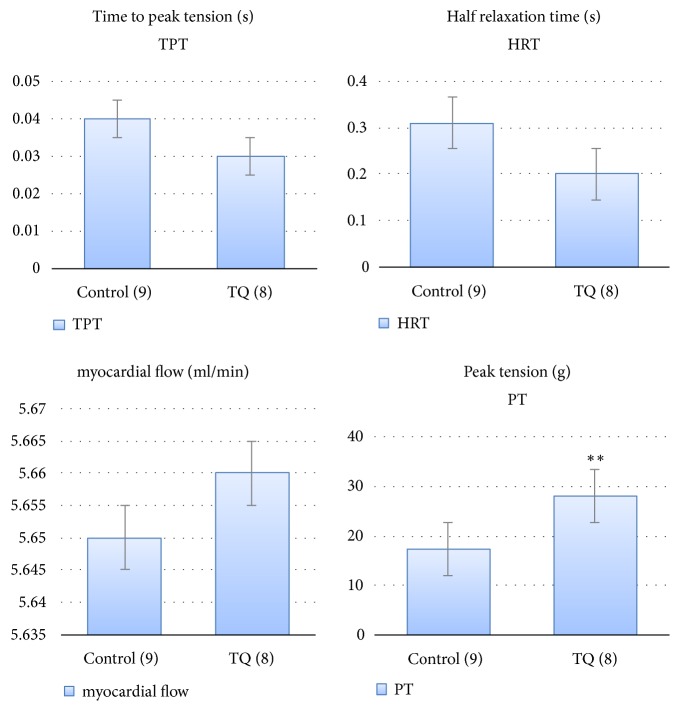
Comparison of the cardiac contractility data of the control and thymoquinone groups. *∗∗* p<0.01.

**Figure 4:**
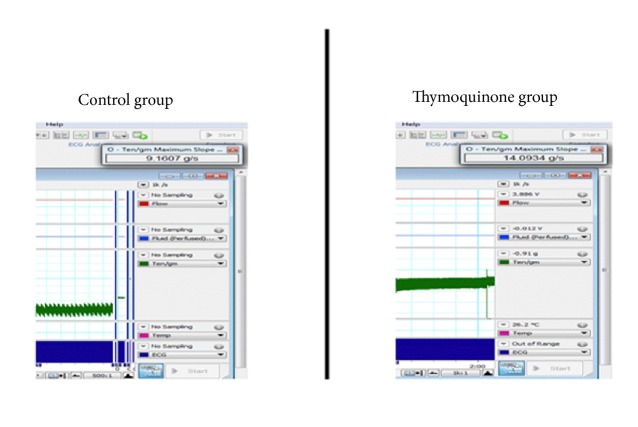
Peak tension recorded from a control rat and a thymoquinone-treated rat.

An inotropic effect was demonstrated by significantly higher peak tension in the TQ group than in the control group (Figures 3 and 4).

There were no significant differences in the time to peak tension, half relaxation time, and myocardial flow rate between the two groups (Figure 3).

## 4. Discussion

The current study demonstrated that long-term administration of TQ at a daily oral dose of 10 mg/kg to adult Wistar albino rats for two months can induce a significant positive baseline inotropic effect on the heart. The baseline positive inotropic effect was clearly evident by increased peak tension in the treatment group compared to the control group. This finding of enhanced peak tension was similarly demonstrated by El-Bahai et al. in rats treated with* Ns* for two months [21]. Yar et al. demonstrated an earlier onset of* Ns*-induced enhanced peak tension of the heart after one month of treatment [22]. TQ is an active ingredient in* Ns*, and the current findings may provide evidence for the involvement of TQ in mediating the positive inotropic action of* Ns*. A positive inotropic effect and enhanced cardiac contractility are desirable therapeutic actions in clinical cardiology [26]. Enhanced cardiac contractility due to pharmacological agents and exercise training was reported in the literature [27]. However, the current available methods are limited by either undesirable side effects or difficulty with treatment compliance, such as that required for exercise training [26]. TQ is a safe supplement, as previously reported by Tauseef Sultan et al. [28].

The mechanism of enhanced cardiac contractility has been studied in the literature, but it remains unclear. Theories included improved Ca^++^ transient and intracellular bioavailability due to upregulation of Ca^++^ transport proteins [29] or increased sensitivity of the contractile proteins to Ca^++^ even in the absence of an enhanced Ca^++^ transient [30]. Enhanced Ca^++^ transient is mostly reflected by augmented contraction of the cardiomyocytes and accelerated relaxation [31]. In catecholamine-induced inotropic effects, increases in peak tension and the maximum rate of tension development are accompanied by decreased half relaxation time and acceleration of the force decline. Therefore, it was proposed that beta adrenergic-induced inotropic effects are probably mediated by an enhanced Ca^++^ transient and upregulation of Ca^++^ transport proteins [32]. However, in the current study, there was no significant difference in the half relaxation time between the two groups. Half relaxation time is an indication of the rate of decline in force and intracellular Ca^++^ bioavailability, also known as a lusitropic effect. Therefore, we could propose that the TQ inotropic effect is mediated by an enhanced sensitivity of contractile proteins to Ca^++^. Likewise, pyruvate-induced inotropic effects showed similar features, and myofilament Ca^++^ sensitization was observed on isolated fibres via potassium contractures [30]. Further studies are recommended to assess Ca^++^ transport and contractile protein sensitivity in TQ-treated cardiomyocytes.

Furthermore, in this study, there was no sign of cardiac hypertrophy, as demonstrated by comparable heart and left ventricle weights and the HW/BW, LVW/BW, and LVW/HW ratios between the two studied groups. In previous studies, long-term oral administration of* Ns* to adult Wistar rats for one or two months yielded increases in heart and left ventricle weights and the relative heart and left ventricle weights-to-body weight ratios (HW/BW, LVW/BW) [21, 22, 33]. The TQ dose and duration in the current study were adjusted to match the corresponding dose of* Ns* that induces positive inotropic and hypertrophic effects. Therefore, TQ may not be a mediating substance in the induction of cardiac hypertrophy, or it may require the presence of other* Ns* ingredients. Furthermore, histological studies to determine the diameter of cardiomyocyte might be needed to confirm this finding as previously demonstrated by Al Asoom et al. [33]. On the other hand, the acquisition of a positive inotropic effect and enhanced contractility without cardiac hypertrophy may be a sufficient and preferable outcome.

Moreover, there was no significant change in myocardial flow rate between the TQ and control groups despite enhanced myocardial contractility. Multiple explanations can be proposed for this phenomenon. One explanation is the lack of myocardial hypertrophy from the observed TQ-induced inotropic effect in contrast to* Ns*-induced cardiac effects, which include cardiac hypertrophy, positive inotropy, chronotropy, and an increased myocardial flow rate [25]. Other explanations include efficient utilization of oxygen by enhanced contractile proteins, efficient oxygen extraction, enhanced synchrony of the regional blood flow, and shortening segments [7, 34]. Further studies are required to explore and confirm the molecular mechanism.

## 5. Conclusions

The present study demonstrated that the administration of oral TQ to adult Wistar albino rats for two months resulted in a positive inotropic effect observed as enhanced baseline peak tension without acceleration of the force decline or increases in the myocardial flow rate. In contrast to long-term* Ns* administration, TQ treatment did not lead to cardiac hypertrophy. The observed cardiac effect of TQ in the current study is promising in the development of new inotropic agents with broad safety ranges and high cost-effectiveness in the field of cardiology.

## Figures and Tables

**Table 1:** Initial and final body weights (IBW, FBW) and weight gain (∆ Wt) of the control and thymoquinone groups.

	Control group	Thymoquinone group
	(N=9)	(N=8)
IBW (g)	222±33.1	239±16.9
FBW (g)	252±35.8	253±23.9
∆ Wt (g)	30.0±11.7	17.1±17.7
∆ Wt%	14±6	10±7

## Data Availability

The datasets used and/or analysed during the current study are available from the corresponding author on reasonable request.

## Abbreviations

TQ: Thymoquinone
HW: Heart weight
BW: Body weight
LVW: Left ventricular weight
*Ns*: * Nigella sativa*
L-NAME: N(*ω*)-nitro-L-arginine methyl ester
IU: International unit
PT: Peak tension
TPT: Time to peak tension
HRT: Half relaxation time
SPSS: Statistical Package for Social Sciences
SD: Standard deviation.
